# Ultrasound-Induced Changes in the Physicochemical and Functional Properties of Whole and Defatted Tiger Nut Flours: Association with Endogenous Lipid Content

**DOI:** 10.3390/foods15193437

**Published:** 2026-09-25

**Authors:** Jing Zhu, Wenhao Hu, Xiaolong Shen, Wenrong Meng, Tiancheng Sheng, Caihong Xu, Fei Lu, Longkun Wu

**Affiliations:** School of Food Science and Technology, Shenyang Normal University, Shenyang 110031, China

**Keywords:** ultrasound, amylose–lipid complex, rheological properties, emulsifying properties, tiger nut flour

## Abstract

Ultrasound treatment can alter the functional properties of flour systems by modifying the organization of starch, protein, lipids, and other matrix components. In this study, whole tiger nut flour (WTF) and defatted tiger nut flour (DTF) were treated with probe ultrasonication at nominal power settings of 0, 300, 600, and 900 W. Pasting behavior, gel rheology, thermal transitions, X-ray diffraction (XRD), protein electrophoretic profiles, intrinsic fluorescence, and emulsifying properties were evaluated to assess how endogenous lipids influence ultrasound-induced changes in tiger nut flour. The response to ultrasound depended on both flour type and the property measured. In WTF, peak viscosity increased at 300 W and decreased at higher power settings, whereas DTF showed a non-monotonic pasting response with partial recovery at 900 W. The storage modulus of WTF gels was highest at 600 W, whereas the rheological response of DTF was comparatively small and non-monotonic. Sonicated WTF exhibited a broad diffraction feature near 2θ ≈ 20°, which is consistent with, but does not confirm, the presence of V-type amylose-containing structures. Differential scanning calorimetry revealed high-temperature endothermic transitions whose origin could not be assigned unambiguously, because starch-related transitions, protein denaturation, water redistribution, and other matrix effects may overlap in whole-flour systems. SDS-PAGE showed no visible change in the molecular-weight distribution of the major extracted proteins. The emulsifying activity and stability of the extracted protein fractions increased up to 600 W and declined at 900 W. Collectively, the results show that endogenous lipids are associated with distinct ultrasound responses in tiger nut flour. However, the present evidence does not directly establish amylose–lipid complex formation, starch–protein complexation, or a specific lipid-mediated protein-protection mechanism; direct structural analyses are required to verify these possibilities.

## 1. Introduction

Tiger nut (*Cyperus esculentus* L.) is a multifaceted tuber crop known to have a good nutritional value with about 40–50% starch, 6–12% high-quality protein, and 15–25% unsaturated fatty acids. It has a high energy density and good processing flexibility, which have made it an attractive plant-based food ingredient over the past few years [1,2]. Despite its attractive nutritional profile, native tiger nut flour is limited in practical applications by several intrinsic constraints: low solubility [3], low emulsifying stability [4], and high hot-paste viscosity [5].

Physical modification methods have garnered widespread attention for the functional property modification of tiger nut flour, owing to their prominent advantages of environmentally friendly processing, high modification efficiency, and absence of chemical reagent residues [4,6]. Among these physical modification techniques, ultrasound treatment exerts its modification effects mainly through the synergistic action of cavitation effect, mechanical shear force, and localized instantaneous high temperature and high pressure [7,8,9,10,11,12]. While ultrasound modification has shown encouraging results for corn, pea, and potato flours [7,9,11], those studies mainly focused on isolated starch or protein systems. Tiger nut flour may present additional complexity because its high endogenous lipid content (15–25%) could contribute an additional lipid-rich phase that may interact with starch and protein during sonication. This complexity may not be fully represented in the aforementioned single- or two-component systems. Moreover, the multiphase interactions induced by ultrasound in whole, native flour systems containing intrinsic lipids remain poorly understood.

The endogenous lipid content of tiger nut flour, mainly oleic and linoleic acids, is particularly noteworthy. These lipids are capable of forming V-type amylose–lipid complexes (ALCs) under certain conditions, a structure that has been shown to affect thermal behavior, crystallinity, and, possibly, protein structure and interfacial behavior [13,14,15]. Previous studies have documented that lipids can form inclusion complexes with amylose in isolated starch systems under thermal or shear conditions [16,17,18]. Non-thermal physical fields such as ultrasound have been reported to promote ALC formation in isolated systems [19,20]. However, it remains unclear how endogenous lipids modulate the structural reorganization of whole tiger nut flour under ultrasound treatment, and to what extent such in situ formed complexes contribute to the overall changes in physicochemical and functional properties. Amylose–lipid complexes have also been used to construct low-glycemic starch-based nanocomposites and hydrogel systems [21].

To address these gaps, we formulated the following testable hypotheses: (i) endogenous lipids in WTF preferentially interact with leached amylose during sonication to form ALCs, thereby competing with starch–protein interactions in DTF; (ii) this lipid-mediated complexation modulates thermal transitions and pasting profiles differently between WTF and DTF.

In order to answer these questions, a comparative model was used in this study with the use of lipid-containing whole tiger nut flour (WTF) and defatted tiger nut flour (DTF); we systematically examined the impact of different nominal ultrasound power (0, 300, 600, and 900 W) on structural organization, thermal properties, rheological properties and emulsifying functionality. X-ray diffraction (XRD), intrinsic fluorescence spectroscopy, SDS-PAGE and rheology were used to explore possible structural and physicochemical changes in the starch–lipid–protein system of tiger nut flour, with particular attention to the potential role of lipids. Because whole-flour matrices are inherently complex, these techniques can provide only indirect and complementary evidence regarding possible molecular interactions. Definitive confirmation of specific complex types would require additional spectroscopic (FTIR, solid-state NMR) and microscopic (SEM/TEM, confocal) analyses, which are identified as critical follow-up studies in Section 3. This work is an attempt to not only provide a theoretical basis for the valorization of tiger nut but also offer novel perspectives on the creation of physically modified, multi-functional plant-based ingredients.

## 2. Materials and Methods

### 2.1. Materials

Dehulled tiger nuts (*Cyperus esculentus* L.) were sourced from the 54th Regiment, Third Division, Xinjiang Production and Construction Corps, China. Refined soybean oil (food grade) was commercially available. An SDS-PAGE reagent kit was bought from Solarbio Science & Technology Co., Ltd. (Beijing, China). All other chemicals, including sodium dodecyl sulfate (SDS), Coomassie Brilliant Blue R-250, bromophenol blue, n-hexane, Tris-HCl, β-mercaptoethanol, glycerol, sodium dihydrogen phosphate monohydrate, disodium hydrogen phosphate (anhydrous), glycine, methanol, glacial acetic acid, sodium hydroxide, and hydrochloric acid, were of analytical grade and obtained from Tianjin Damao Chemical Reagent Co., Ltd. (Tianjin, China).

### 2.2. Preparation of Raw Materials

Tiger nuts were shelled, ground, and sieved through an 80-mesh screen (aperture 0.177 mm) to obtain WTF, representing the lipid-containing sample. DTF was subsequently prepared from WTF.

Defatting was conducted following a modified procedure based on the method described by Cui et al. [6]. Briefly, WTF was mixed with n-hexane at a ratio of 1:3 (*w*/*v*) and stirred using a magnetic stirrer for 1.5 h. The mixture was then centrifuged at 8000× *g* for 15 min. The extraction process was repeated four times to ensure sufficient lipid removal. Because defatting causes particle agglomeration due to lipid removal and moisture redistribution, the resulting precipitate was gently disaggregated and passed through a 0.5 mm sieve to obtain a free-flowing powder, yielding the DTF. This coarser sieving was necessary to avoid excessive mechanical compression of the defatted particles; importantly, the final particle size distributions showed no significant difference between WTF (D50 = 78.5 ± 2.1 μm, D10 = 32.4 ± 1.8 μm, D90 = 156.3 ± 5.2 μm, span = 1.58 ± 0.06) and DTF (D50 = 82.3 ± 1.9 μm, D10 = 35.1 ± 2.0 μm, D90 = 162.7 ± 4.8 μm, span = 1.55 ± 0.05) (*p* > 0.05), confirming that the different sieve specifications did not result in divergent particle size distributions. The residual lipid content of DTF was determined by Soxhlet extraction with n-hexane and found to be 0.82 ± 0.15% (*w*/*w*, dry basis), confirming a lipid removal efficiency of approximately 94.7% based on the initial lipid content of WTF (15.6 ± 0.8%, *w*/*w*, dry basis). All prepared flour samples were stored at 4 °C for subsequent use. The moisture contents of WTF and DTF were 8.4 ± 0.3% and 9.1 ± 0.4% (*w*/*w*), respectively, determined by oven-drying at 105 °C to constant weight.

### 2.3. Ultrasound Treatment

Ultrasound treatment was carried out using a probe-type ultrasonic processor (SCIENTZ-IID, Ningbo Scientz Biotechnology Co., Ltd., Ningbo, China) operating at a fixed frequency of 20 kHz, fitted with a 20 mm diameter titanium alloy probe (effective radiating area: 3.14 cm^2^). The actual acoustic power was determined calorimetrically according to the method of Gogate et al. [22]: the probe was immersed at a depth of 2.0 cm in 100 g of distilled water, and the temperature rise recorded at 5 s intervals over the initial 60 s was used to calculate the effective power as P = m·Cp·(dT/dt). The nominal settings of 0, 300, 600, and 900 W corresponded to effective power densities of approximately 0, 9.8, 19.6, and 29.4 W/cm^2^, and, for a 10 min treatment in pulse mode (5 s on/3 s off), to energy inputs of 58.8, 117.6, and 176.4 J/g, respectively. The calorimetric measurements were used to characterize the operating conditions; the nominal instrument settings were retained as the primary treatment descriptors for the flour suspensions. WTF and DTF were separately dispersed in distilled water at a solid-to-liquid ratio of 1:5 (*w*/*v*) and treated at 0 W (untreated control), 300 W, 600 W, and 900 W. All treatments were performed in an ice-water bath with continuous temperature monitoring using the PT100 sensor supplied with the processor; the maximum temperature recorded during treatment was 28.0 ± 1.5 °C. After sonication, samples were freeze-dried (−50 °C, 10 Pa, 48 h), ground, passed through an 80-mesh sieve, and stored at 4 °C until analysis.

### 2.4. Determination of Pasting Properties

A Rapid Visco Analyzer (RVA) (Techmaster, Perten Instruments, Hägersten, Sweden) was employed to measure the pasting properties of the flour samples. For each test, 3 g of flour (adjusted to 14% moisture basis) was mixed with 25 g of distilled water. The suspension was incubated at 50 °C for 1 min, then heated to 95 °C at a rate of 12.2 °C/min, held at 95 °C for 2.5 min, and subsequently cooled to 50 °C at a rate of 11.8 °C/min. The viscosity profile was recorded throughout the entire test cycle.

### 2.5. Determination of Rheological Properties

The rheological properties of the pastes obtained after RVA analysis were evaluated using a HAAKE RheoStress 6000 rheometer (Thermo Fisher Scientific, Waltham, MA, USA) (parallel-plate geometry, 40 mm diameter, 1 mm gap). After loading, the sample was equilibrated at 25 °C for 5 min. The storage modulus (G′) and loss modulus (G″) were then obtained by a frequency sweep between 1 and 10 Hz at a constant strain of 1% (within the linear viscoelastic region, verified by a preliminary strain sweep). The full frequency-sweep data, including G′, G″, and tan δ versus frequency, are provided in Figure S2 (Supplementary Material). The modulus data were fitted with the power-law equations G′ = G′_1_·ωᵃ and G″ = G″_1_·ωᵇ, where G′_1_ and G″_1_ are the moduli at 1 Hz (Pa), ω is the angular frequency (rad/s), and a and b are dimensionless exponents. The loss tangent was fitted analogously as tan δ = (tan δ)_1_·ωᶜ.

### 2.6. Determination of Thermal Properties

A Differential Scanning Calorimeter (DSC-250, TA Instruments, New Castle, DE, USA) was used to measure the thermal properties of the samples, using a method reported by Gu et al. [23] with slight modifications. Approximately 5 mg of sample was accurately weighed into an aluminum pan, mixed with 15 µL of distilled water, and hermetically sealed. An empty hermetically sealed aluminum pan was used as the reference. The sealed sample pan was equilibrated at 4 °C overnight prior to testing and then heated from 40 °C to 200 °C at a constant rate of 10 °C/min. The upper temperature limit of 200 °C was chosen to cover the full range of endothermic events that may occur in a whole-flour system, including starch-related transitions, possible amylose–lipid dissociation events, and higher-temperature transitions that cannot be excluded in defatted flour (e.g., protein denaturation) [24,25]. Because these events may overlap, all transitions are reported as apparent values: the heat flow curve was used to determine the apparent endothermic enthalpy (ΔH) and the characteristic transition temperatures (To, Tp, and Te). Apparent enthalpy values were normalized to the dry mass of flour. Because the measured endotherms were not resolved into component-specific contributions, these values were not interpreted as starch-gelatinization enthalpies.

### 2.7. X-Ray Diffraction (XRD) Analysis

XRD was used to study the crystalline structural changes in the samples. An X-ray diffractometer (MiniFlex 600, Rigaku, Ltd., Tokyo, Japan) with 40 kV and 30 mA was used in the analysis. The samples were scanned at a scan rate of 1.2°/min in a 2θ angular range between 5° and 40°. Relative crystallinity (RC) was calculated by the method of Nara and Komiya [26] using OriginPro 2021 software: the diffraction peak area and the total diffraction area over the 2θ range of 5–40° were determined after baseline correction, and RC was expressed as the percentage of crystalline peak area relative to the total area.

### 2.8. Protein Extraction and Characterization

Proteins were extracted from both WTF and DTF samples. For WTF, defatting with n-hexane (1:3 *w*/*v*, 1.5 h, repeated twice) was performed prior to protein extraction to remove interfering lipids; for DTF, which had already undergone the defatting procedure described in Section 2.2, no additional defatting step was required. The defatted sample was suspended in ten times its weight of distilled water. The suspension was adjusted to pH 9.5 with 1.0 mol/L NaOH and then stirred at 45 °C for 2 h. This was centrifuged and the supernatant was taken. The process of protein precipitation was carried out by changing the pH of the supernatant to 4.5 with 1.0 mol/L HCl. The precipitate was dried, redissolved and freeze-dried. SDS-PAGE was used to determine the molecular weight distribution of the extracted proteins with a 5% stacking gel and a 12% separating gel.

### 2.9. Emulsifying Properties

The emulsifying properties were measured according to the method described below. The sample was dissolved in phosphate-buffered saline (PBS, 0.2 mol/L, pH 7.0) to a final concentration of 0.2 mg/mL. Soybean oil was then added to the solution at a 1:3 (*v*/*v*) oil-to-aqueous-phase ratio. The mixture was homogenized at 10,000 rpm for 1 min to form an emulsion. Aliquots of the emulsion were taken immediately (0 min) and after standing for 10 min, and the absorbance at 500 nm was recorded. The emulsifying activity index (EAI, m^2^/g) and emulsifying stability index (ESI, min) were calculated according to the following equations:

(1) $$\mathrm{EAI}=\frac{2.303\times A_{0}\;\text{×}\;N}{\varphi \;\text{×}\;L\;\text{×}\;C\;\text{×}\;10,000}$$

(2) $$\mathrm{ESI}=\frac{A_{0}\;\text{×}\;10}{A_{0}-A_{10}}$$

where A_0_ and A_10_ are the absorbances measured at 500 nm immediately after emulsion formation (0 min) and after 10 min of standing, respectively (dimensionless); N is the dilution factor (N = 100, dimensionless); L is the path length of the cuvette (L = 1 cm); φ is the volume fraction of the oil phase (φ = 0.25, dimensionless); and C is the protein concentration before emulsification (mg/mL).

### 2.10. Fluorescence Spectroscopy

Protein samples were dissolved in 0.2 mol/L PBS (pH 7.0) to a final concentration of 0.2 mg/mL. Intrinsic fluorescence spectra of the samples were recorded using a fluorescence spectrophotometer (F-4500, Hitachi, Tokyo, Japan). The excitation wavelength was set at 290 nm, and the emission spectrum was scanned from 300 nm to 450 nm. The instrument parameters were set as follows: excitation and emission slit width of 5.0 nm, photomultiplier tube (PMT) voltage of 600 V, and scan speed of 300 nm/min. The fluorescence signal of PBS buffer blank was subtracted from all sample measurements.

### 2.11. Statistical Analysis

All experiments were conducted using three independent sample preparations. Each analytical measurement was performed in duplicate when applicable, and the duplicate measurements were averaged before statistical analysis. The results are presented as mean ± standard deviation (SD), with *n* = 3 independent preparations. Data were first assessed for normality using the Shapiro–Wilk test and for homogeneity of variances using Levene’s test. The assumptions of normality and homoscedasticity were considered acceptable when *p* > 0.05. For the prespecified key response variables, two-way analysis of variance (ANOVA) was performed to evaluate the effects of flour type (whole tiger nut flour and defatted tiger nut flour), nominal ultrasound power (0, 300, 600, and 900 W), and their interaction. For the comparison of the eight treatment combinations presented in Table 1, Table 2 and Table 3, one-way ANOVA followed by Tukey’s honestly significant difference (HSD) post hoc test was used. Differences were considered statistically significant at *p* < 0.05. All analyses were performed with SPSS 22.0 (IBM, Corp., Armonk, NY, USA), and the charts were generated using OriginPro 2021 software.

## 3. Results and Discussion

### 3.1. Effects of Ultrasound on Pasting Properties

The pasting characteristics of WTF and DTF were assessed using a Rapid Visco Analyzer (Perten Instruments, Hägersten, Sweden); the resulting viscosity curves are presented in Figure 1, and the corresponding parameters are summarized in Table 1. Notably, the untreated DTF control exhibited a higher peak viscosity (768 cP) than WTF (493 cP). The higher peak viscosity of DTF may reflect changes in matrix composition and water accessibility after defatting. A contribution from reduced lipid-associated restriction of starch swelling is possible, but it was not directly demonstrated [27]. For WTF, peak viscosity increased from 493 ± 11.53 cP in the untreated control to 673 ± 20.60 cP at 300 W and then declined progressively at higher power levels. The initial increase may be due to cavitation-induced disruption of the starch granule surface, which increases water absorption and amylose leaching [8,23]. The subsequent drop at 600 W and 900 W may be attributed to excessive mechanical shear, which disrupts starch molecular chains and thereby lowers swelling capacity and paste viscosity [28,29]. Final viscosity followed a similar pattern, rising from 725 ± 17.58 cP in the control to 827 ± 27.22 cP at 300 W and then decreasing to 743 ± 21.28 cP and 683 ± 25.53 cP at 600 W and 900 W, respectively. In contrast, DTF showed a non-monotonic response: peak viscosity rose from 768 ± 23.58 cP for the control to 1104 ± 38.74 cP at 300 W, decreased to 930 ± 30.20 cP at 600 W, and then increased again to 1034 ± 35.04 cP at 900 W. Trough and final viscosities showed similar trends, with recovery occurring at the highest power level. Peak time varied from 4.87 to 5.07 min. Within each flour type, the ultrasound-treated samples were not clearly separated from their respective controls according to the post hoc grouping, although the overall comparison also reflected differences between WTF and DTF. The viscosity recovery observed for DTF at 900 W cannot be explained by starch degradation alone. It may reflect competing changes in starch organization, particle structure, protein conformation, and water accessibility. Because no direct interaction measurement was performed, the contribution of starch–protein association cannot be determined [30]. It must be emphasized that direct evidence for such protein–starch complexes (e.g., FTIR amide I band shifts, confocal microscopy, or surface plasmon resonance) was not obtained in this study, and this possibility remains an avenue for future research. This interpretation is consistent with the rheological response of DTF (Section 3.3), in which the storage modulus at 900 W (159.02 ± 21.88 Pa) was slightly higher than at 600 W (130.07 ± 22.38 Pa), interrupting the otherwise decreasing trend. No such viscosity recovery was observed for WTF at 900 W. This difference between the two flours may be related to the presence of endogenous lipids: The XRD and DSC data are compatible with, but do not demonstrate, ultrasound-promoted amylose–lipid interactions in WTF (Section 3.2 and Section 3.4). If such interactions occurred, they could reduce the level of free amylose and limit amylose–protein contacts, offering one possible explanation for the absence of a viscosity rebound in WTF. Furthermore, at all nominal ultrasound power levels, WTF consistently showed lower peak and final viscosities than DTF, which may reflect differences in matrix composition, water accessibility, and starch–lipid interactions; the present data do not establish the formation of V-type complexes [27].

Overall, these findings suggest that ultrasound modifies pasting behavior through two competing processes: moderate and high nominal power settings were associated with different changes in paste development, potentially reflecting competing effects on particle structure, water accessibility, starch organization, and protein conformation. The contribution of protein–starch interactions cannot be determined from the present data. The response of WTF was compatible with, but did not establish, a possible contribution from amylose–lipid interactions. These mechanistic interpretations should be regarded as hypotheses, as the present data provide only indirect, comparative evidence.

### 3.2. Thermal Properties Analysis

The DSC results are summarized in Table 2, and the thermograms are presented in Figure S1. The apparent endothermic enthalpy of WTF was 25.16 ± 2.74 J/g in the untreated control and 24.44 ± 3.45 J/g at 300 W, increasing to 73.10 ± 8.50 and 72.57 ± 8.15 J/g at 600 and 900 W, respectively. The corresponding peak temperatures increased from approximately 107 °C in the control and 300 W samples to approximately 143–145 °C at the two higher power settings. In DTF, the mean apparent enthalpy decreased from 164.45 ± 12.25 J/g in the untreated control to 65.58 ± 7.50 J/g at 900 W. The peak temperatures of DTF ranged from 158.83 to 170.64 °C, without a monotonic relationship with nominal ultrasound power. Because the enthalpy values were obtained from whole-flour samples and may include overlapping events, comparisons with enthalpies reported for isolated starch should be treated as qualitative only [31].

Several of the observed peak temperatures, particularly those in the high-temperature region, were above the range commonly reported for starch gelatinization under excess-water conditions. They are therefore reported as apparent endothermic transitions rather than assigned exclusively to starch gelatinization. Because the samples contained starch, proteins, lipids, and other flour components, overlapping thermal events and differences in water accessibility may have contributed to the measured profiles. However, the present measurements do not establish the contribution of any individual component.

The relatively high enthalpy values, particularly those of DTF, also require caution. Reporting enthalpy per gram of dry flour does not, by itself, explain values substantially above those commonly reported for isolated starch gelatinization. Interpretation requires verification of the integration limits, baseline selection, sample-mass normalization, and pan integrity. Consequently, the observed enthalpy changes cannot be used alone to infer either disruption of starch crystallinity or formation of additional ordered structures.

The broad XRD feature near 2θ ≈ 20° in sonicated WTF provides a separate indication of altered structural organization, as discussed in Section 3.4. Nevertheless, neither this feature nor the DSC results identify a specific amylose–lipid complex or its thermal dissociation. The thermal results therefore indicate differences between WTF and DTF after ultrasound treatment, while the molecular origin of the observed transitions remains unresolved. Further measurements using controlled hydration conditions, appropriately rated sealed pans, and separated flour fractions would help distinguish the contributions of the individual matrix components.

### 3.3. Rheological Properties Analysis

The rheological parameters of gels prepared from both WTF and DTF are summarized in Table 3. For all samples across the entire frequency sweep range (1–10 Hz), the storage modulus (G′) was consistently greater than the loss modulus (G″), indicating that all gels exhibited predominantly elastic, solid-like behavior (frequency-sweep curves are shown in Figure S2, Supplementary Material). Previous studies have shown that V-type ALCs can increase the cross-linking density of starch-based gel networks and thereby elevate G′ [32]; if ALCs were indeed formed in sonicated WTF, a similar contribution to gel elasticity could be expected, although this interpretation remains hypothetical.

For WTF, G′ increased from 300 to 600 W and declined slightly at 900 W, with the highest value at 600 W (289.37 ± 53.01 Pa). Because the 900 W value showed high variation (265.35 ± 101.43 Pa, CV ≈ 38%), differences among the three treated groups should be interpreted with caution. The higher mean G′ at 600 W may reflect changes in starch organization, protein conformation, or lipid-associated matrix structure. If amylose–lipid structures were formed, they could contribute to the observed gel response; however, their presence and structural role were not demonstrated in this study [32]. This is consistent with the results of Putseys et al. [16], who found that adding ALCs affects the gelling behavior of starch suspensions.

For DTF, G′ and G″ showed a non-monotonic response. Both values reached their lowest mean values at 600 W and increased at 900 W. The bubbles and shear forces created during ultrasonic treatment can disrupt the hydrogen bonds and supramolecular structures of starch, weakening the gel network [33]. Here, the term “chain breakage” does not imply cleavage of covalent bonds in the starch polymers; rather, it refers to disruption of hydrogen bonds and supramolecular organization. Changes in starch molecular weight or covalent-chain integrity were not measured; therefore, the present results do not establish whether molecular chain scission occurred. The mean G′ value increased at 900 W relative to 600 W, although the treatment groups did not necessarily differ significantly according to the post hoc grouping. The increase at 900 W may reflect competing effects of ultrasound on starch organization, protein conformation, particle structure, and water accessibility. Because no direct interaction analysis was performed, the contribution of starch–protein association cannot be determined. Cao et al. [30] reported that microwave treatment affected oat starch–protein complex characteristics and starch digestion; Gul et al. [34] reported that high-intensity ultrasound altered the functional and rheological properties of sesame proteins.

### 3.4. Structural Analysis (XRD)

Figure 2 shows the XRD patterns of the WTF and DTF samples. For all ultrasonically treated samples, the intensity of the native A-type crystalline diffraction peaks was lower than that of the untreated controls, indicating a reduction in the apparent X-ray-crystalline order of the flour samples. Quantitatively, the relative crystallinity (RC) of the WTF untreated control was 8.90%, which decreased to 7.24% at 300 W and further to 2.00% at 900 W; similarly, the DTF untreated control showed 8.90% RC, declining to 4.02% at 300 W and 2.00% at 900 W. The identical RC values of both untreated controls reflect the similar baseline crystallinity of the native starch in the two flours; the DTF control was derived from the same WTF batch, and the defatting process (n-hexane extraction, 4 cycles) did not significantly alter the starch crystalline structure, as confirmed by the comparable XRD patterns. Separate XRD measurements were performed for both WTF and DTF untreated controls, and the superimposed patterns are presented in Figure 2 (gray traces). In the WTF samples, a broad diffraction feature was observed at 2θ ≈ 20° after ultrasonic treatment. This scattering angle is consistent with the reported characteristic reflection of V-type amylose–lipid structures [35], and its appearance is also roughly consistent with the pasting and thermal data in Table 1 and Table 2. However, a single broad peak at 2θ ≈ 20° is insufficient to definitively identify V-type crystalline structures in such a complex flour matrix, because proteins and other components may contribute to the diffraction pattern. We therefore describe this feature as “suggestive of” rather than “confirming” V-type ALC formation. If ALCs were formed, the mechanical shear and cavitation generated during ultrasonic treatment would have disintegrated swollen starch particles and increased the availability of linear starch chains able to interact with endogenous lipids [13,36]. Similar phenomena have been reported for pea starch, where ultrasonic treatment was accompanied by the appearance of a V-type diffraction feature attributed to interactions between starch and endogenous lipids [37]. The broad feature may reflect low structural order, peak overlap, or contributions from other flour components. Its molecular origin cannot be determined from the present XRD pattern alone. At 300 W, the A-type peaks weakened more markedly in DTF (RC from 8.90% to 4.02%) than in WTF (8.90% to 7.24%), consistent with more extensive disruption of crystalline order in the absence of lipids. The relative-crystallinity data indicated power-dependent changes in both flour types. However, the available measurements do not allow the conversion of A-type domains into V-type complexes to be demonstrated.

Further analysis compared the relative crystallinity of the samples to quantify the changes in starch crystalline structure under ultrasound. For the DTF group, ultrasound treatment resulted in a marked, power-dependent decrease in relative crystallinity, consistent with previous studies on ultrasound modification of after defatting [38]; this decrease can be attributed to cavitation loosening and disrupting the internal granule structure [39]. The apparent relative crystallinity decreased in the reported DTF samples as nominal ultrasound power increased. In WTF, the reported values also decreased from the untreated control to 300 and 900 W. These results indicate power-dependent changes in the apparent crystalline order of the flour samples, but they do not demonstrate the conversion of A-type domains into V-type amylose–lipid complexes.

### 3.5. Protein Structure and Function Analysis

The SDS-PAGE profiles of proteins extracted from WTF and DTF are shown in Figure 3. The major bands occurred at approximately 16, 19–21, and 29–42 kDa, with no obvious changes in their migration positions after ultrasound treatment. Thus, no substantial alteration in the molecular-weight distribution of the major extracted proteins was detected under the electrophoretic conditions used. Similar observations have been reported for ultrasound-treated soy proteins [40,41]. However, unchanged band positions do not exclude limited molecular changes or alterations in protein conformation and association.

The lower staining intensity observed for DTF-900 W was treated as a qualitative observation. Band intensity can be influenced by protein recovery, sample loading, and staining conditions, and was therefore not used as evidence of protein aggregation or starch–protein complexation. Furthermore, the extraction procedure and denaturing conditions used for SDS-PAGE limit its ability to characterize interactions present in the original flour matrix.

The intrinsic fluorescence spectra are presented in Figure 4. Fluorescence intensity increased after ultrasound treatment relative to the untreated controls, with the highest intensity observed at the lowest nonzero power setting, followed by a decrease at higher settings. WTF-derived protein fractions exhibited higher fluorescence intensity than DTF-derived fractions at corresponding power settings, while the emission maximum remained approximately unchanged. These observations are consistent with changes in the local environment or quenching behavior of aromatic residues [42]. However, fluorescence intensity alone cannot distinguish partial unfolding, reassociation, or differences in the composition and recovery of the extracted protein fractions.

The emulsifying properties are shown in Figure 5. Both EAI and ESI reached their highest reported values at 600 W and decreased at 900 W. Power-dependent changes in protein structure and functionality under high-intensity ultrasound, including declines at high power, have been reported for other plant protein systems [43]. Improvements in the emulsifying properties of plant proteins after ultrasound treatment have been related to increased molecular flexibility and surface hydrophobicity [44,45]. For the WTF-derived protein fraction, EAI increased from 87.82 m^2^/g in the untreated control to 234.91 m^2^/g at 600 W, while ESI increased from 11.36 to 21.22 min. These changes corresponded to increases of approximately 167.5% and 86.8%, respectively. At 600 W, the corresponding values for the DTF-derived fraction were 144.63 m^2^/g and 15.19 min. The reported decline between 600 and 900 W was more pronounced in DTF than in WTF.

Interpretation of the WTF–DTF comparison must also account for differences in sample preparation. WTF was defatted after ultrasound treatment and before protein extraction, whereas DTF was defatted before ultrasound treatment. Subsequent alkaline extraction and isoelectric precipitation may further influence the properties of the recovered fractions. Accordingly, the measurements characterize the extracted proteins and do not directly establish the emulsifying behavior of the original flours or a protective effect of endogenous lipids during sonication.

Overall, the extracted protein fractions showed power-dependent changes in fluorescence and emulsifying properties without obvious changes in the migration positions of the major SDS-PAGE bands. The differences between WTF and DTF are compatible with effects of flour composition and processing history, but they do not establish competitive amylose–lipid binding, starch–protein complexation, or lipid-mediated protection against aggregation. The interaction pathways illustrated in Figure 6 should therefore be regarded as hypotheses for future testing.

## 4. Conclusions

This study shows that ultrasound treatment modified the techno-functional properties of tiger nut flour in a manner dependent on the nominal power setting and flour type, with changes observed in starch- and protein-related measurements. The optimal power level was property-dependent: 600 W gave the highest mean G′ for WTF gels and the highest emulsifying indices of the extracted protein fractions from both flour types, whereas 300 W gave the highest peak viscosity in both flours. In WTF, moderate ultrasonication was accompanied by an increased apparent endothermic enthalpy and peak temperature and by the appearance of a broad diffraction feature at 2θ ≈ 20°. While these data are consistent with ALC formation, they do not provide definitive structural confirmation, which would require complementary techniques such as FTIR or solid-state NMR in future studies; consequently, all assignments in this manuscript should be regarded as tentative. The higher mean G′ at 600 W was associated with improved elastic response, but the molecular basis of this response remains unresolved. Simultaneously, ultrasound treatment at 600 W was associated with fluorescence changes and improved emulsifying properties, which may reflect alterations in protein conformation or extractability. Endogenous lipids are associated with distinct ultrasound responses: WTF showed lower paste viscosity than DTF at all power levels and a smaller decline in emulsifying properties at 900 W. Whether these differences arise from amylose–lipid complexation, from altered partitioning of mechanical energy in the suspension, or from other lipid-related matrix effects cannot be distinguished with the present data, and no conclusion is drawn regarding possible lipid-associated modulation of protein-related functionality. For applications requiring higher peak viscosity in WTF, the 300 W treatment may be more appropriate.

## 5. Limitations and Future Directions

The present study has several limitations that should be acknowledged. First, the proposed mechanism of ultrasound-induced ALC formation is supported only by indirect evidence derived from XRD, DSC, rheological measurements, and comparative analyses of WTF and DTF. Although these findings are consistent with ALC formation, they do not provide direct structural confirmation. Additional techniques such as FTIR, solid-state NMR, Raman spectroscopy, or microscopic analyses would considerably strengthen the proposed model. Second, the hypothesis that endogenous lipids competitively interact with amylose and thereby reduce possible changes in protein extractability or conformation remains hypothetical. The current experimental data do not directly demonstrate competitive binding or protein protection; surface hydrophobicity assays, confocal microscopy, or molecular docking studies would be required to validate this competition mechanism. Third, the ultrasound treatment is described primarily in terms of nominal power (W). Although nominal power, treatment time, and the solid-to-liquid ratio are reported, the probe amplitude, batch volume, and acoustic-field distribution in the flour suspensions were not fully characterized; future studies should consider calorimetric power mapping or computational fluid dynamics modeling to fully describe the energy input. Fourth, the practical implications of these modifications for specific food applications are discussed only briefly. Future work should include proof-of-concept trials in model food systems (e.g., gluten-free bread, plant-based yogurt, or salad dressings) to validate the technological benefits of 600 W-treated tiger nut flour. The performance of the treated flours in formulated foods remains to be evaluated.

## Figures and Tables

**Figure 1:**
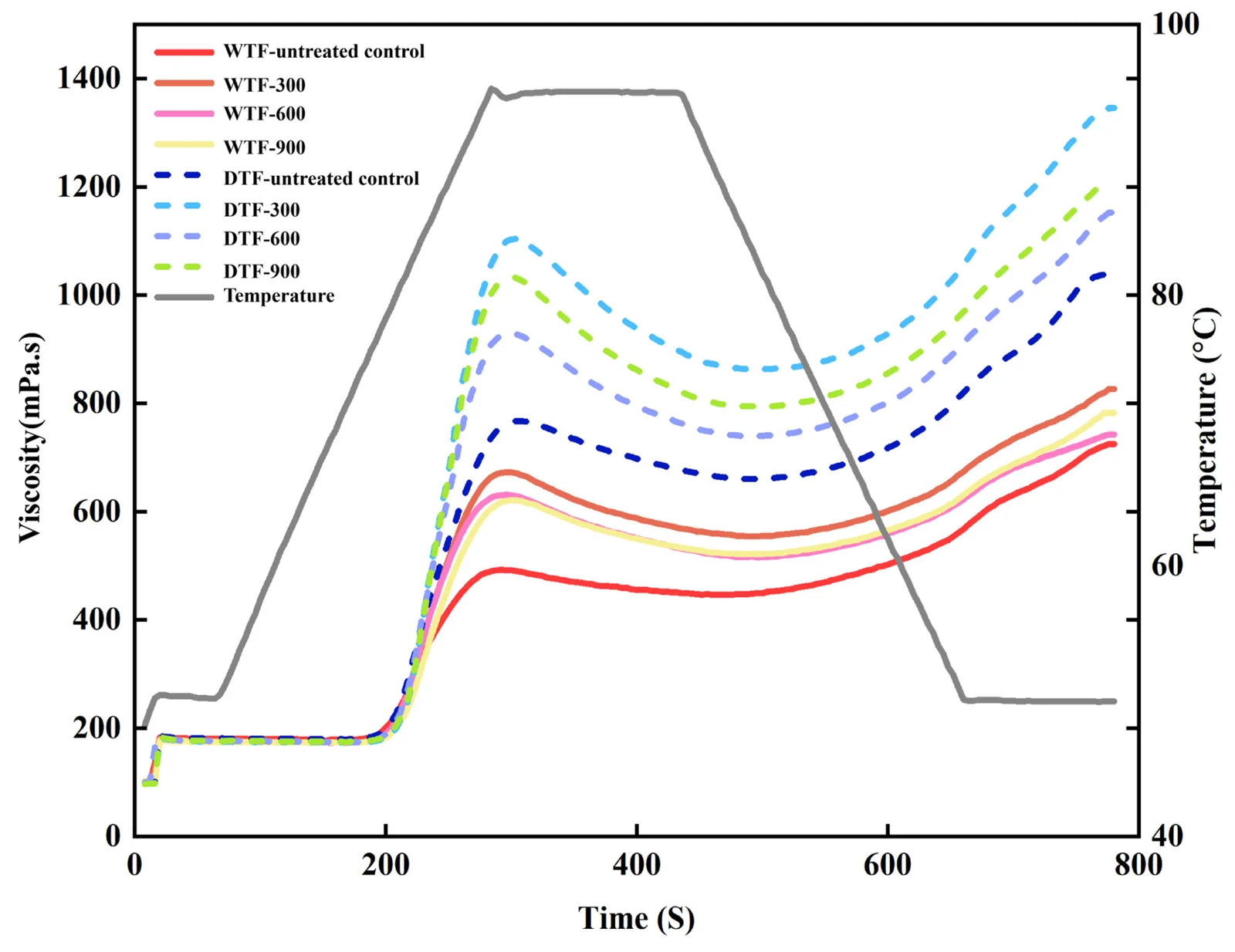
RVA pasting curves of whole tiger nut flour (WTF) and defatted tiger nut flour (DTF) under various nominal ultrasound power levels (0, 300, 600, and 900 W). Viscosity is expressed in centipoise (cP; 1 cP = 1 mPa·s).

**Figure 2:**
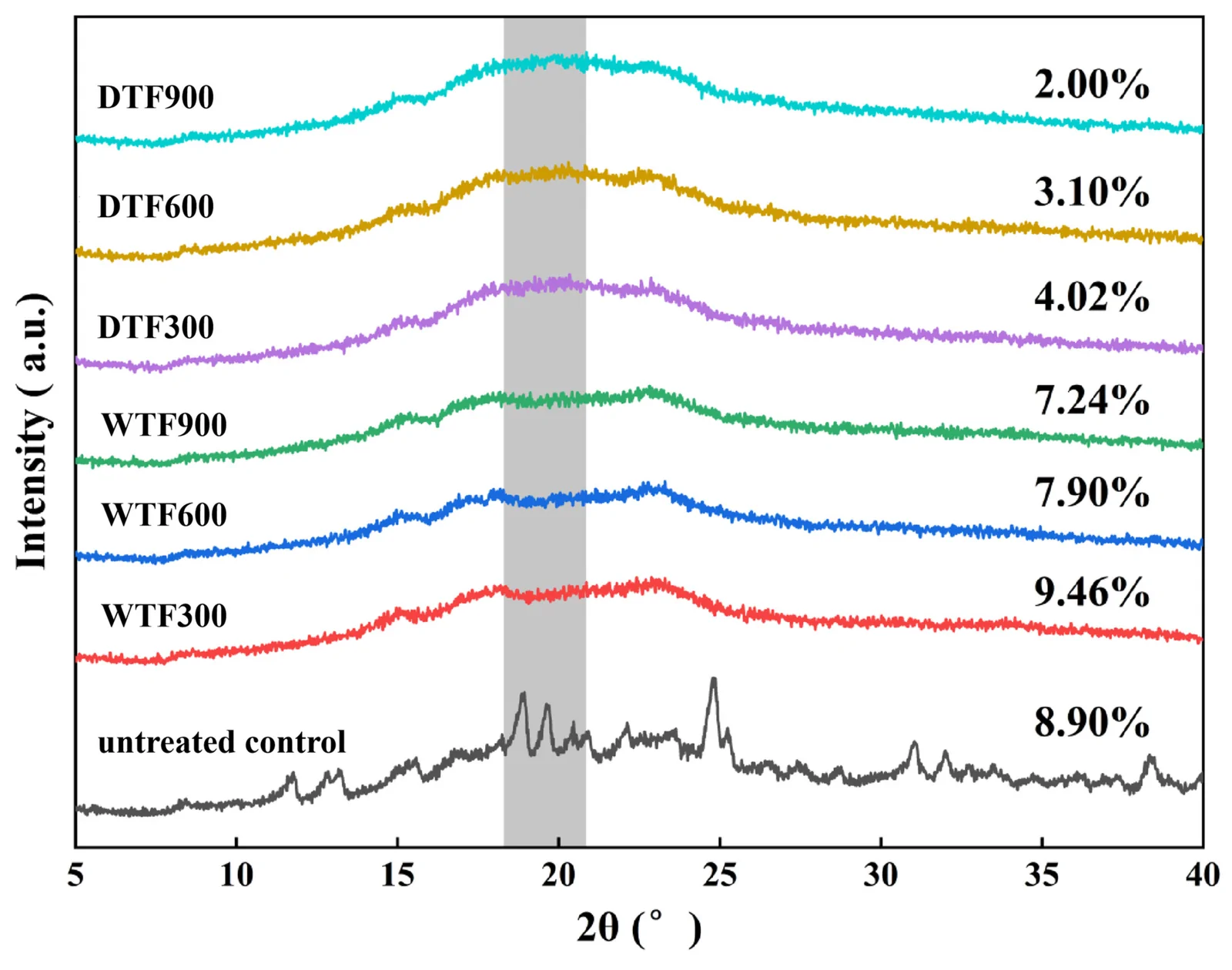
X-ray diffraction (XRD) patterns of untreated and ultrasound-treated WTF and DTF. The gray-shaded band marks the region near 2θ ≈ 20° where sonicated WTF samples exhibited a broad diffraction feature consistent with, but not confirmatory of, V-type amylose–lipid structures.

**Figure 3:**
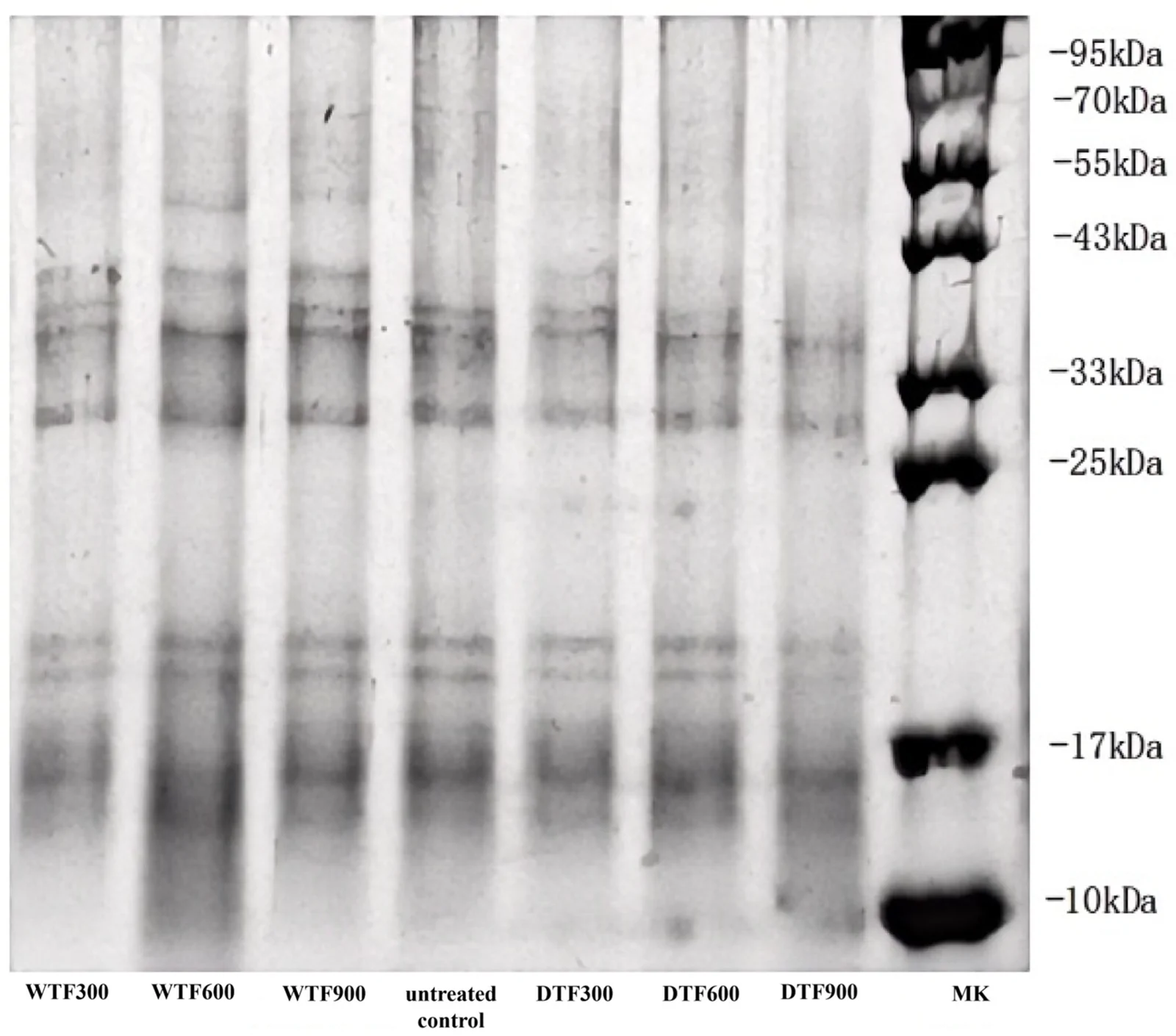
Sodium dodecyl sulfate–polyacrylamide gel electrophoresis (SDS-PAGE) profiles of proteins extracted from WTF and DTF under different nominal ultrasound power conditions. MK: molecular weight marker.

**Figure 4:**
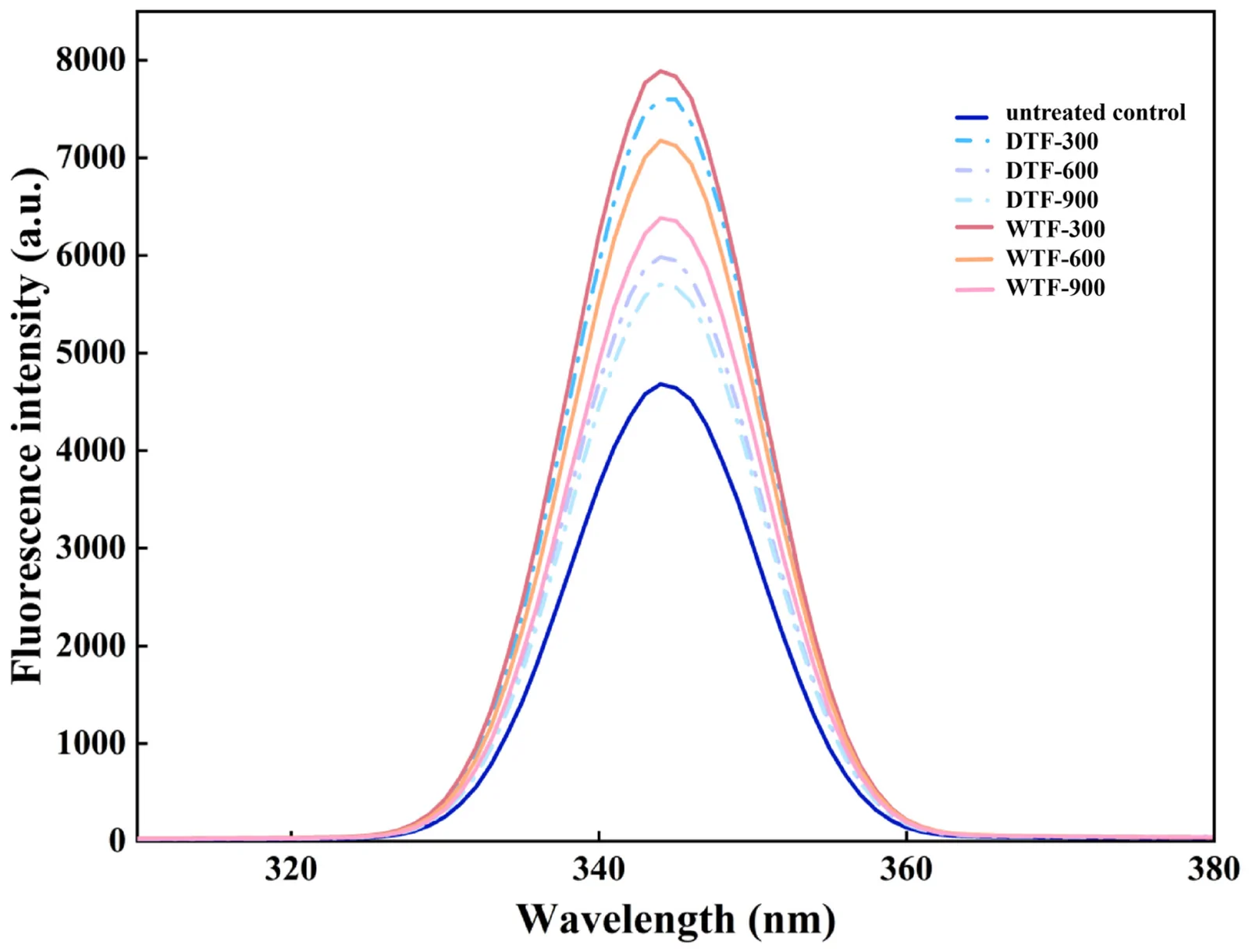
Intrinsic fluorescence spectra of proteins extracted from WTF and DTF under different nominal ultrasound power conditions.

**Figure 5:**
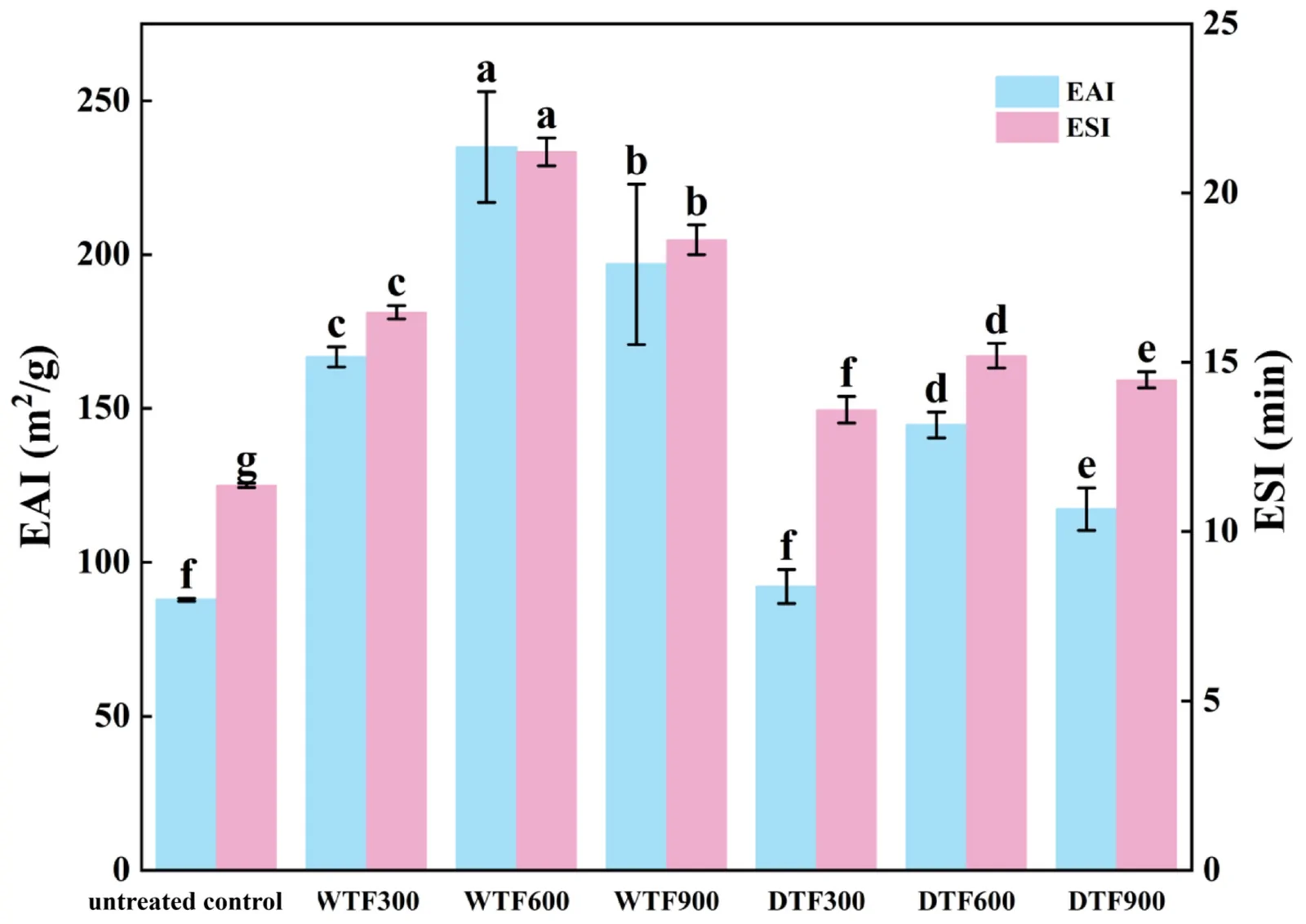
Emulsifying activity index (EAI) and emulsifying stability index (ESI) of WTF and DTF proteins under different nominal ultrasound power conditions. Different lowercase letters indicate significant differences among the eight treatment combinations according to Tukey’s HSD test (*p* < 0.05).

**Figure 6:**
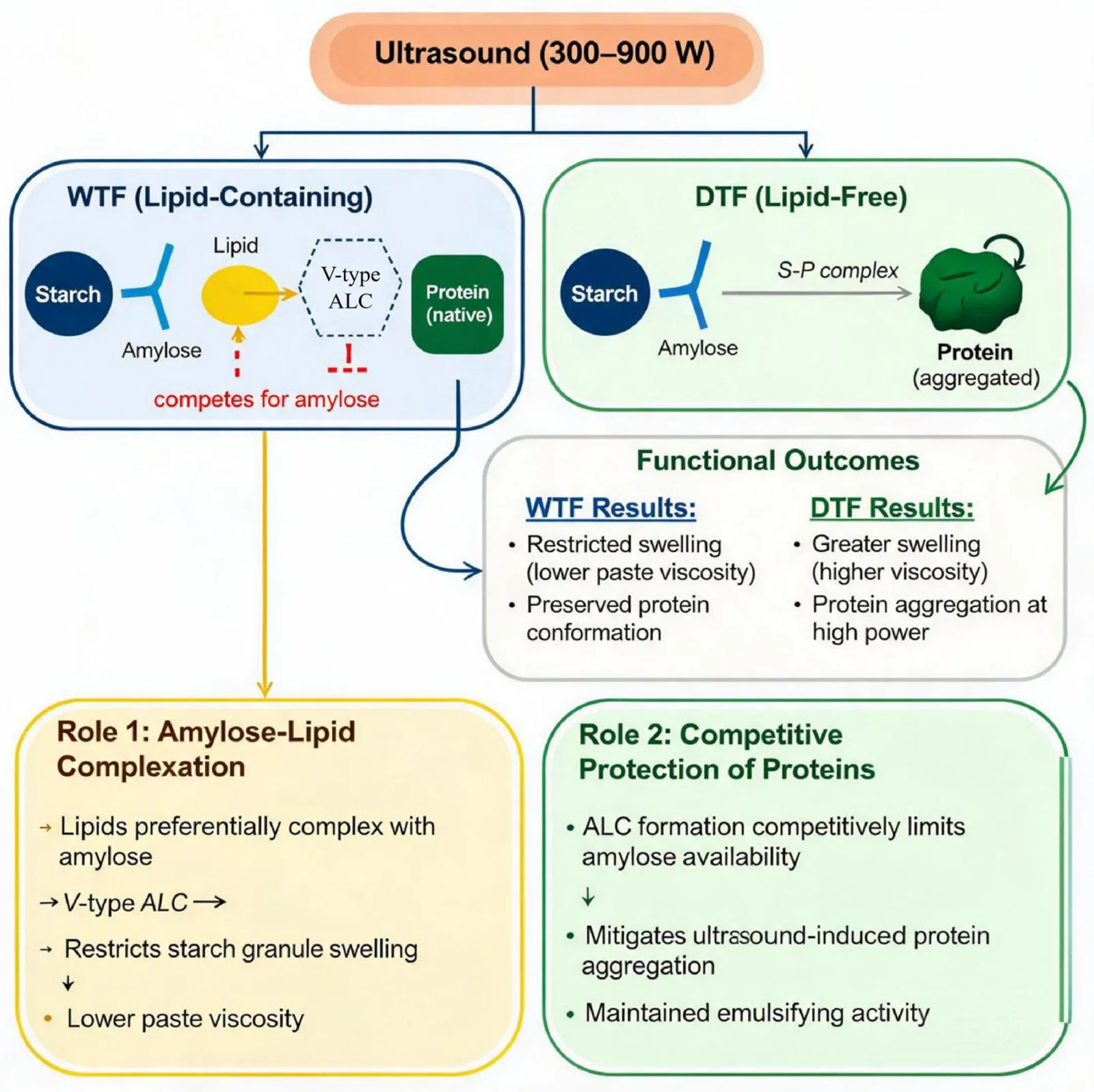
Schematic model illustrating two hypothetical roles of endogenous lipids during ultrasound treatment of tiger nut flour. Ultrasound (300–900 W) was applied to whole tiger nut flour (WTF, lipid-containing) and defatted tiger nut flour (DTF, lipid-free), as indicated by the solid blue arrows. Solid arrows (→) denote transformation or causal sequences (e.g., lipid–amylose interaction leading to V-type amylose–lipid complex (ALC) formation, and amylose–protein interaction leading to the starch–protein (S–P) complex); dashed red arrows (⇢) denote competitive interactions, i.e., competition between lipid and protein for available amylose; downward arrows (↓) denote a consequent decrease or restriction (e.g., restricted starch granule swelling and reduced amylose availability); and curved arrows indicate the corresponding functional outcomes. Left pathway: in WTF, lipids may interact with leached amylose to form V-type amylose–lipid complexes, which could restrict granule swelling and contribute to the lower paste viscosity relative to DTF. Right pathway: such interactions could reduce the availability of free amylose for contacts with proteins, indirectly limiting starch–protein interactions under sonication. In DTF, the substantially reduced lipid content may alter the opportunity for starch–protein association, although this was not directly measured, which is one possible explanation for the partial recovery of paste viscosity and storage modulus at 900 W. All lipid-mediated pathways shown are hypothetical; none is directly demonstrated by the XRD, DSC, rheological, or SDS-PAGE data presented here.

**Table 1:** Pasting properties of whole tiger nut flour (WTF) and defatted tiger nut flour (DTF).

Sample	Pasting Temperature (°C)	Peak Viscosity (cP)	Peak Time (min)	Trough Viscosity (cP)	Breakdown Viscosity (cP)	Final Viscosity (cP)	Setback Viscosity (cP)
WTF untreated control	81.60 ± 0.30 ^a^	493 ± 11.53 ^e^	4.87 ± 0.041 ^b^	477 ± 7.94 ^f^	16 ± 3.60 ^d^	725 ± 17.58 ^ef^	248 ± 12.12 ^c^
WTF-300	80.80 ± 0.40 ^ab^	673 ± 20.60 ^d^	4.93 ± 0.034 ^ab^	555 ± 13.0 ^e^	118 ± 6.56 ^c^	827 ± 27.22 ^d^	272 ± 10.54 ^c^
WTF-600	80.75 ± 0.45 ^ab^	632 ± 16.50 ^d^	4.93 ± 0.06 ^ab^	516 ± 11.53 ^ef^	116 ± 7.55 ^c^	743 ± 21.28 ^de^	227 ± 13.53 ^c^
WTF-900	80.40 ± 0.60 ^b^	622 ± 24.27 ^d^	5.00 ± 0.08 ^ab^	522 ± 17.09 ^ef^	100 ± 9.85 ^c^	683 ± 25.53 ^ef^	161 ± 15.10 ^d^
DTFuntreated control	80.75 ± 0.35 ^ab^	768 ± 23.58 ^c^	5.07 ± 0.065 ^a^	661 ± 16.52 ^d^	107 ± 8.54 ^c^	1036 ± 28.00 ^c^	375 ± 17.00 ^b^
DTF-300	81.50 ± 0.30 ^ab^	1104 ± 38.74 ^a^	5.07 ± 0.05 ^a^	864 ± 24.51 ^a^	240 ± 14.53 ^a^	1346 ± 44.00 ^a^	482 ± 20.52 ^a^
DTF-600	80.70 ± 0.50 ^ab^	930 ± 30.20 ^b^	4.93 ± 0.06 ^ab^	740 ± 21.00 ^c^	190 ± 11.53 ^b^	1153 ± 36.59 ^b^	413 ± 18.52 ^b^
DTF-900	81.50 ± 0.40 ^ab^	1034 ± 35.04 ^b^	5.00 ± 0.07 ^ab^	795 ± 22.52 ^b^	239 ± 13.53 ^a^	1214 ± 39.03 ^b^	419 ± 21.52 ^b^

Values are expressed as mean ± standard deviation (*n* = 3). Different lowercase letters within the same column indicate significant differences among the eight treatment combinations according to Tukey’s HSD test (*p* < 0.05).

**Table 2:** Thermal properties of whole tiger nut flour (WTF) and defatted tiger nut flour (DTF).

Sample	Apparent Transition Enthalpy (J/g, Dry Basis)	To (°C)	Tp (°C)	Te (°C)	ΔT (°C)
WTF untreated control	25.16 ± 2.74 ^d^	105.79 ± 1.75 ^e^	106.75 ± 2.20 ^c^	124.15 ± 2.45 ^e^	18.36 ± 1.15 ^b^
WTF-300	24.44 ± 3.45 ^d^	101.71 ± 2.20 ^e^	106.97 ± 2.25 ^c^	126.67 ± 2.75 ^e^	24.96 ± 0.85 ^a^
WTF-600	73.10 ± 8.50 ^c^	130.04 ± 3.25 ^d^	143.00 ± 4.10 ^b^	155.25 ± 4.45 ^cd^	25.21 ± 1.80 ^a^
WTF-900	72.57 ± 8.15 ^c^	141.22 ± 3.55 ^c^	144.58 ± 3.80 ^b^	154.09 ± 3.90 ^cd^	12.87 ± 1.05 ^c^
DTF untreated control	164.45 ± 12.25 ^ab^	150.91 ± 4.80 ^bc^	158.83 ± 5.20 ^a^	179.70 ± 6.10 ^ab^	28.79 ± 2.10 ^a^
DTF-300	148.80 ± 10.60 ^ab^	151.28 ± 4.50 ^bc^	161.33 ± 4.95 ^a^	167.39 ± 5.70 ^bc^	16.11 ± 1.80 ^bc^
DTF-600	144.80 ± 9.80 ^ab^	169.45 ± 5.15 ^a^	170.64 ± 5.30 ^a^	186.45 ± 6.25 ^a^	17.00 ± 1.90 ^bc^
DTF-900	65.58 ± 7.50 ^c^	158.31 ± 4.20 ^b^	164.96 ± 4.55 ^a^	180.96 ± 4.85 ^ab^	22.65 ± 1.60 ^ab^

To, Tp, and Te represent the onset, peak, and conclusion temperatures of the observed endothermic transitions, respectively. ΔT = Te − To. Because the samples were whole-flour systems, these parameters should not be interpreted exclusively as starch-gelatinization parameters. Different lowercase letters within the same column indicate significant differences among the eight treatment combinations according to Tukey’s HSD test (*p* < 0.05).

**Table 3:** Rheological properties of whole tiger nut flour (WTF) and defatted tiger nut flour (DTF).

Sample	G′_1_ (Pa)	a	G″_1_ (Pa)	b	(tan δ)_1_	c
WTF untreated control	151.78 ± 27.46 ^b^	0.06 ± 0.01 ^a^	32.22 ± 6.40 ^b^	0.10 ± 0.01 ^ab^	0.21 ± 0.01 ^a^	0.03 ± 0.01 ^b^
WTF-300	257.13 ± 24.50 ^a^	0.05 ± 0.00 ^a^	47.74 ± 3.51 ^a^	0.08 ± 0.01 ^c^	0.19 ± 0.01 ^b^	0.02 ± 0.01 ^b^
WTF-600	289.37 ± 53.01 ^a^	0.06 ± 0.01 ^a^	51.67 ± 5.81 ^a^	0.07 ± 0.01 ^c^	0.19 ± 0.02 ^ab^	0.02 ± 0.01 ^a^
WTF-900	265.35 ± 101.43 ^a^	0.07 ± 0.01 ^a^	47.06 ± 15.37 ^a^	0.09 ± 0.02 ^bc^	0.19 ± 0.01 ^b^	0.03 ± 0.01 ^b^
DTF untreated control	150.96 ± 46.80 ^b^	0.28 ± 0.38 ^a^	25.27 ± 6.89 ^b^	0.10 ± 0.01 ^ab^	0.170 ± 0.01 ^b^	0.03 ± 0.01 ^b^
DTF-300	142.95 ± 23.39 ^b^	0.06 ± 0.01 ^a^	25.42 ± 1.49 ^b^	0.10 ± 0.01 ^ab^	0.18 ± 0.02 ^b^	0.04 ± 0.02 ^b^
DTF-600	130.07 ± 22.38 ^b^	0.05 ± 0.00 ^a^	23.98 ± 6.29 ^b^	0.11 ± 0.02 ^a^	0.18 ± 0.02 ^b^	0.06 ± 0.01 ^b^
DTF-900	159.02 ± 21.88 ^b^	0.07 ± 0.01 ^a^	28.06 ± 3.67 ^b^	0.09 ± 0.01 ^abc^	0.18 ± 0.00 ^b^	0.03 ± 0.01 ^b^

G′_1_, G″_1_, and (tan δ)_1_ represent the storage modulus, loss modulus, and loss tangent at 1 Hz, respectively. a, b and c are the frequency power law exponents of storage modulus (G′), loss modulus (G″) and loss tangent (tan δ), respectively. Values are mean ± standard deviation (*n* = 3). Different lowercase letters in the same column indicate significant differences (*p* < 0.05) among samples.

## Data Availability

The original contributions presented in this study are included in the article/Supplementary Material. Further inquiries can be directed to the corresponding authors.

## Supplementary Materials

The following supporting information can be downloaded at https://www.mdpi.com/article/10.3390/foods15193437/s1, Figure S1: DSC thermograms of whole tiger nut flour (WTF) and defatted tiger nut flour (DTF) under different ultrasound power levels (0, 300, 600, and 900 W). Samples (approximately 5 mg) were mixed with 15 µL of distilled water in hermetically sealed aluminum pans and heated from 40 °C to 200 °C at 10 °C/min (Section 2.6 of the main text). WTF-600 W and WTF-900 W showed additional high-temperature endothermic transitions (>140 °C), and DTF samples exhibited elevated apparent transition temperatures. Because the measured endotherms were not resolved into component-specific contributions, these transitions are reported as apparent events and are not assigned to specific molecular origins (see Section 3.2 of the main text); Figure S2: Frequency-sweep data of the pastes obtained after RVA analysis (Section 2.5 of the main text). A: Storage modulus (G′, squares) and loss modulus (G″, circles) as functions of angular frequency (1–10 Hz). B: Loss tangent (tan δ) as a function of angular frequency. Different colors denote different treatment combinations; the same color denotes the same sample in bothpanels. Table S1: Results of two-way ANOVA (significance levels) for the effects of flour type (F1), ultrasound power (F2), and their interaction (F1×F2) on the pasting, thermal, rheological, and emulsifying properties of WTF and DTF.
