# Supplementary material for: Ultrasound-Induced Changes in the Physicochemical and Functional Properties of Whole and Defatted Tiger Nut Flours: Association with Endogenous Lipid Content
Source: Foods. 2026 Sep 25;15(19):3437. doi: 10.3390/foods15193437 (PMC13664465; doi:10.3390/foods15193437)
Supplement: Supplementary file 1 [file foods-15-03437-s001.zip › foods-4498866-supplementary.pdf]

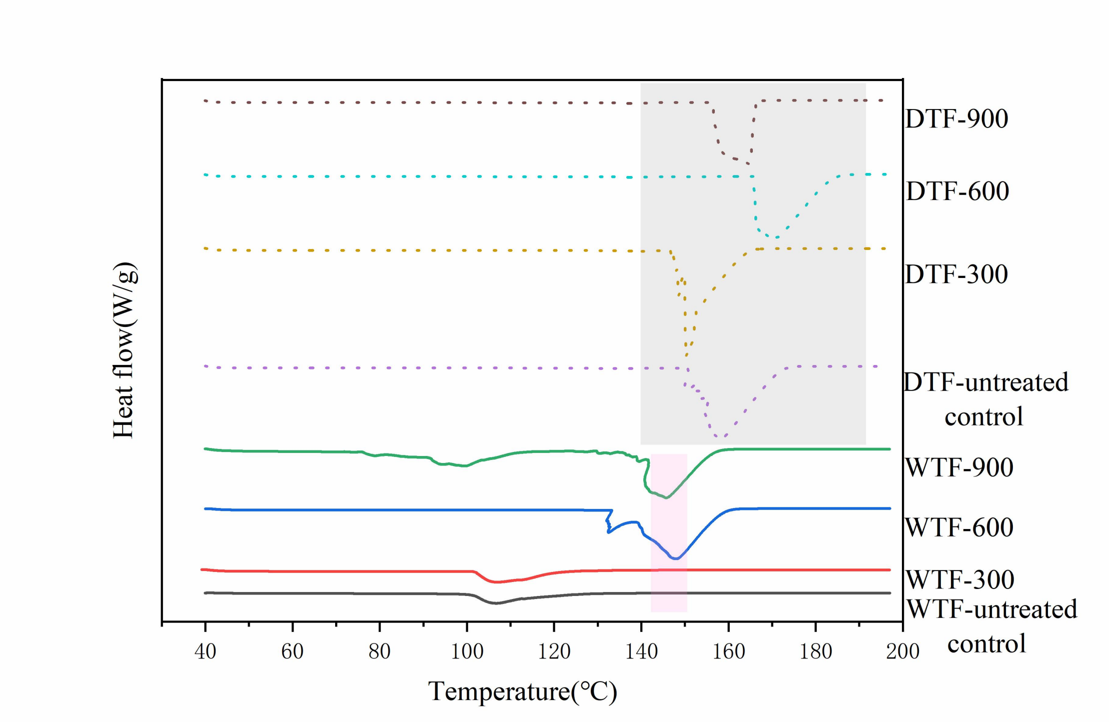

Figure S1. DSC thermograms of whole tiger nut flour (WTF) and defatted tiger nut flour (DTF) under different ultrasound power levels (0, 300, 600, and 900 W). Samples (approximately 5 mg) were mixed with 15 µL of distilled water in hermetically sealed aluminum pans and heated from 40 °C to 200 °C at 10 °C/min (Section 2.6 of the main text). WTF-600 W and WTF-900 W showed additional high-temperature endothermic transitions (>140 °C), and DTF samples exhibited elevated apparent transition temperatures. Because the measured endotherms were not resolved into component-specific contributions, these transitions are reported as apparent events and are not assigned to specific molecular origins (see Section 3.2 of the main text).

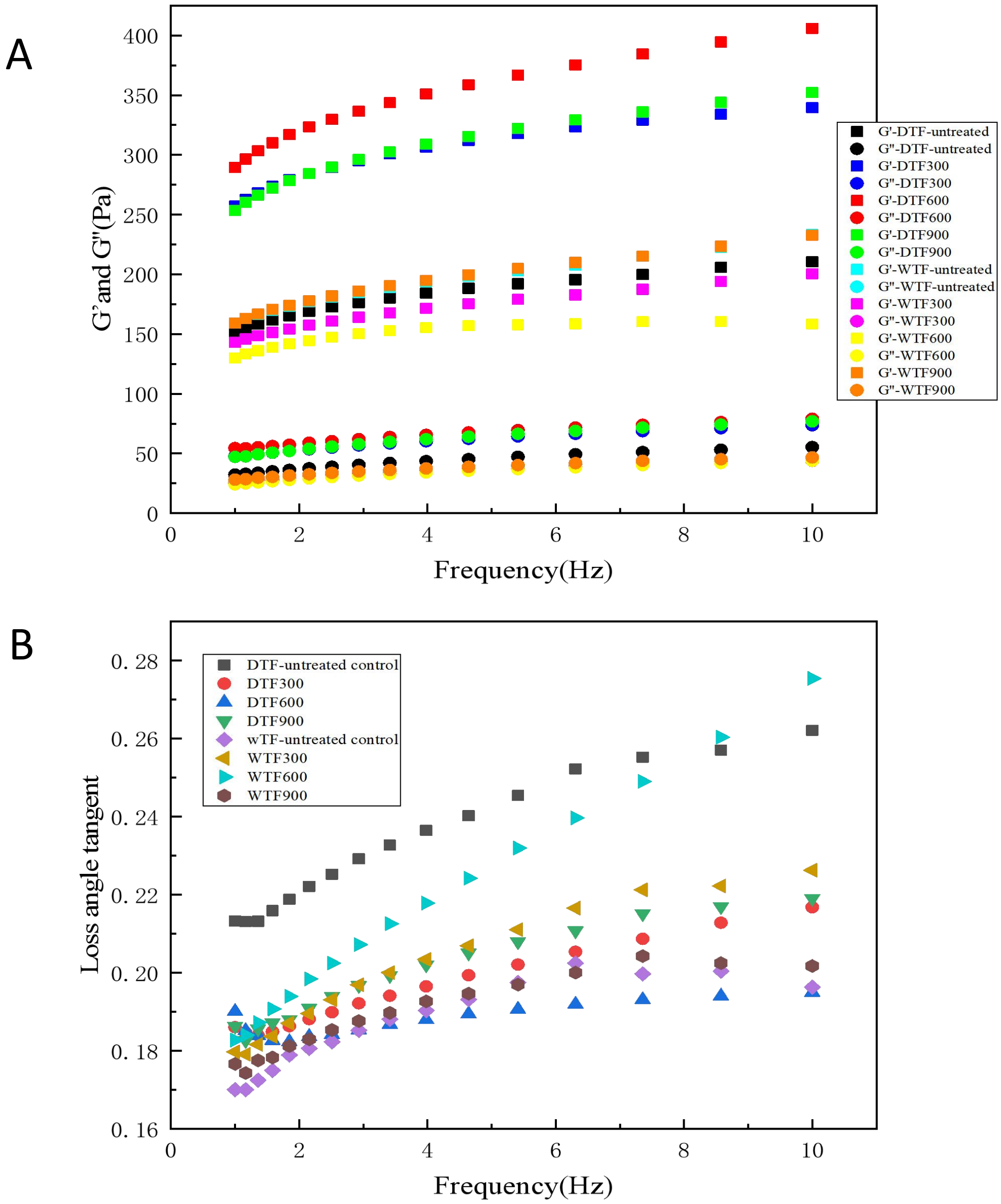

Figure S2. Frequency-sweep data of the pastes obtained after RVA analysis (Section 2.5 of the main text). A: Storage modulus (G′, squares) and loss modulus (G″, circles) as functions of angular frequency (1–10 Hz). B: Loss tangent (tan δ) as a function of angular frequency. Different colors denote different treatment combinations; the same color denotes the same sample in both panels.

Table S1. Results of two-way ANOVA (significance levels) for the effects of flour type (F1), ultrasound power (F2), and their interaction (F1×F2) on the pasting, thermal, rheological, and emulsifying properties of WTF and DTF.

Sample	F1	F2	F1×F2
Gelatinization Temperature（℃）	ns	ns	**
Peak Viscosity（cP）	***	***	***
Peak Time（min）	*	ns	*
Trough Viscosity（cP）	***	***	***
Breakdown Viscosity（cP）	***	***	***
Final Viscosity（cP）	***	***	***
Setback Viscosity（cP）	***	***	***
ΔHgel（J/ g d.b）	***	***	***
To-gel（℃）	***	***	***
Tp-gel（℃）	***	***	***
Te-gel（℃）	***	***	***
ΔT（℃）	ns	***	***
G1’（Pa）	***	*	*
a	ns	ns	ns
G2”（Pa）	***	ns	*
b	**	ns	ns
(Tan δ)1	**	ns	ns
c	**	ns	**
EAI	***	***	ns
ESI	***	***	***

To, Tp, and Te represent the onset, peak, and conclusion temperatures of the observed endothermic transition(s), respectively; ΔT = Te − To. Because the samples were whole-flour systems, these parameters should not be interpreted exclusively as starch-gelatinization parameters. G′1 and G″1 are the storage and loss moduli at 1 Hz, respectively; a, b, and c are the frequency power-law exponents of G′, G″, and tan δ, respectively. EAI and ESI denote the emulsifying activity index and the emulsifying stability index, respectively. F1: flour type (WTF vs. DTF); F2: nominal ultrasound power (0, 300, 600, and 900 W). Statistical analysis was based on three independent sample preparations (Section 2.11 of the main text). * $p < 0.05$; ** $p < 0.01$; *** $p < 0.001$; ns: not significant.
